# Microbial Flora of Root Canals of Pulpally-infected Teeth: Enterococcus faecalis a Prevalent Species

**DOI:** 10.5681/joddd.2009.007

**Published:** 2009-03-16

**Authors:** Esrafil Balaei Gajan, Mohammad Aghazadeh, Rahib Abashov, Amin Salem Milani, Zohreh Moosavi

**Affiliations:** ^1^Lecturer, Department of Community Dentistry, Faculty of Dentistry, Tabriz University of Medical Sciences, Tabriz, Iran; ^2^Assistant Professor, Department of Microbiology, Faculty of Medicine, Tabriz University of Medical Sciences, Tabriz, Iran; ^3^Professor, Head of the Department of Microbiology, School of Biology, Baku State University, Baku, Azerbaijan; ^4^Assistant Professor, Department of Endodontics, Faculty of Dentistry, Tabriz University of Medical Sciences, Tabriz, Iran; ^5^Laboratory Assistant, Faculty of Dentistry, Tabriz University of Medical Sciences, Tabriz, Iran

**Keywords:** Endodontic therapy, Enterococcus faecalis, failure

## Abstract

**Background and aims:**

The aim of this study was to determine the microorganisms prevalent in the necrotic dental pulp and root canals of unsuccessfully treated teeth.

**Materials and methods:**

The present study was conducted on 150 single-rooted teeth of patients referring to a dental clinic. Sampling was performed by placing a sterile paper point in the canal for 60 s. Bacterial samples were evaluated by a microbiological technique specific for anaerobic species, used for isolation and identification of sampled strains.

**Results:**

From the 150 samples taken, 101 were from necrotic pulps (primary infection) and 49 were from the teeth with an unsuccessful endodontic treatment (secondary infection).

**Conclusion:**

Enterococcus faecalis was a prevalent species in the failed root canals evaluated.

## Introduction

It has been established that bacteria initiate pulpal and periapical infections. ^1, 2^ The success of endodontic treatment is directly related to the decrease in the number of root canal microorganisms. Colonizing microorgan-isms result in pulpal and periapical diseases. ^3^ treatment involves the treatment of both primary and secondary infections in the root canal system. Primary infected root canals are untreated canals, into which the microorganisms have gained access to colonize the pul-pal tissue, resulting in dysfunction. ^4^ Secondary infection in the root canal occurs due to the failure of endodontic treatment and presence of bacterial infection in the root canal system. ^5, 6^ Several studies have investigated the microflora of root canal system infections. In primary root canal infections, necrotic pulpal tissue has revealed polymicrobial flora with an average of 4-7 intra-canal species, which are often Gram-negative anaerobics. ^7- 12^ Several studies have shown obligatory anaerobic bacte-ria in root canal infections, which comprise 90% of all bacterial species. ^8, 13^

Studies have reported various species in the pulp of necrotic teeth and, therefore, one or multiple species of bacterial pathogens can be isolated from an infected root canal.^12^ Studies have also demonstrated the presence of facultative aerobic bacteria in the oral cavity, but obligatory anaerobics have not been isolated. ^7,12^

Recent studies investigating bacteria in teeth with an unsuccessful restoration have revealed a certain group of microorganisms including Gram-positive facultative aerobics, especially Enterococcus faecalis. ^6,8, 11-13^ The purpose of the present study was to evaluate the presence of bacteria and spores in the untreated and unsuccessfully-treated root canals of human teeth using culture techniques.

## Materials and Methods

### Case selection

This study was conducted on 150 single-rooted teeth of patients referring to the Dental Clinic of Tabriz University of Medical Sciences between October 2007 and September 2008. Medical and dental histories were taken as part of the routine at the clinic. Patients with a systemic disease or those who had taken antibiotics in the last three months were excluded from the study. Bacterial samples were evaluated by means of an advanced microbiological technique specific for anaerobic species, which was used for isolation and identification of sampled strains.

### Sampling

Sampling was carried out according to the asepsis instructions described previously.^1^ The teeth were isolated with rubber dam. Two-step access cavity preparation was accomplished using sterile burs under water spray. Sterile saline was used where rinsing was necessary. All coronal restorations, posts, and carious lesions were removed. The teeth were rinsed using 30% hydrogen peroxide and 2.5% sodium hypochlorite for 30 s, and 5% sodium thiosulfate was used as the deactivating solution. All the instruments used during access cavity preparation and afterwards were sterile. In the case of a treated canal, canal filling material was removed with the use of Gates Glidden drills and endodontic files without the use of any chemicals. Canals were rinsed with sterile saline to remove the remnants of filling material and debris, and to moisten the canals before sampling. Working length of the canal was determined radiographically using a #20 K-file. The root canal was prepared with a #20 K-file 0.5 mm short of the radiographic working length without any rinsing. Sampling was performed by placing a sterile paper point in the canal for 60 s. During the sampling procedure, nitrogen gas was pumped into the canal orifice. Following removal from the canal, the paper point was immediately put into an intermediate medium containing 3 mL of sterile reduced transport liquid and sent to the microbiology laboratory within 15 min. The maximum time between collecting the samples and starting laboratory procedures was 4 h.

### Isolating and detection of species

In an anaerobic setting in the laboratory, tubes containing transport medium were shaken for 60 s in the mixer. Ten series of 1:104 dilutions of anaerobic unreduced broth were prepared and 50 µL of each was added to the following media: 5% fibrin FAA bovine blood, 0.001% w/v nalidixic acid, 0.025% w/v nalidixic acid and vancomycin, 0.075% w/v neomycin for anaerobics, 5% Colombia agar and fibrin bovine blood (OXOID, Hampshire, UK) for aerobics and 100 µg/mL of Sabouraud’s agar with chloramphenicol for spores. To culture anaerobics, plates were incubated at 37°C under the pressure of 10% H_2_, 10% CO_2_, and 80% N_2_ for 2, 5, and 14 days, respectively. Colombia agar plate was incubated at 37°C for 2 days under aerobic conditions. Sabouraud’s agar plate was kept at room temperature for 5 days. Following incubation, the plates were tested and the colonies were cultured unspecifically on FAA-blood plate to obtain pure cultures. Colonies were selected based on appearance for further evaluation. On the obtained pure culture, Gram staining and catalase production tests were performed and their gaseous need was provided through a 2-day aerobic and anaerobic incubation period. Bacterial colonies were cultured to be identified and purified. Anaerobics were identified using phase contrast microscopy, morphotyping, Gram staining, and biochemical tests with enzymes-based kits (API C AUX, API Staph, Rapid ID 32, Bio Merieux SA, Marcy Etoile, France). Facultative anaerobics and aerobics were identified using Gram staining, micromorphology, colony morphology, growth in specific media, and enzyme-based biochemical kits (Rap IDSIR ad ANA 11 System, IDS. API Strep). Additional tests including fluorescence under ultraviolet light at 366 nm, 3% bovine erythrocyte hemagglutination, lactose fermentation using 4-methyl-β-galactosidease fluorogenic substrate, and determining trypsin-like activity using synthetic fluorogenic peptide were performed for Gram-negative black-pigmented anaerobes.

## Results

Out of 150 samples, 101 were taken from necrotic pulps (primary infection) and 49 had an unsuccessful endodontic treatment (secondary infection). Clinical and radiographic evaluation of the teeth revealed conditions including previous history of pain (40 cases), acute pain (28 cases), sensitivity to percussion (32 cases), tooth mobility (6 cases), swelling (26 cases), bloody canal (36 cases), pus formation (21 cases), hemorrhage (8 cases), clear drainage (7 cases) , and periapical radiolucency (26 cases). Vitality tests showed non-vital pulps in all teeth. In teeth that had been treated endodontically, weak obturation and radiographic signs of apical periodontitis in 19 cases and recurrent caries in 34 cases were present. In 8 secondary infection cases, coronal restorations were not present, and from the 41 cases with coronal restorations, 11 teeth showed microleakage.

In the cultured plates, 35 teeth had only one species, 44 teeth had two species, and 46 teeth showed multimicrobial infection involving three or more species from each root canal. With regard to primary endodontic infection, 17 teeth had only one species, 40 teeth had two species and 34 teeth had multimicrobial infection, involving three or more species. No species was detected in the 10 remaining teeth. Each canal had five species at most.

A total of 197 isolated culturable species were identified from root canals evaluated. Of these, 85 species (43.1%) were obligatory anaerobes and 104 species (52.8%) were facultative anaerobes. Eight species (4.1%) were identified as fungi. 68.9% of isolated species were Gram positive and 27% were Gram negative. 62.4% of species collected from primary necrotic pulps were Gram positive and 31.2% were Gram negative. 77% of species collected from failed endodontically-treated teeth were Gram positive and 23% Gram negative.

Peptostreptococcus was the most prevalent species followed by Streptococcus, Porphyromonas, and Enterococcus faecalis. Strains of P. provetti, S. sanguis, S. salivarius, P. endodontalis, and especially E. faecalis were prevalent in the unsuccessfully-treated root canals. C. albicans, Veillonella spp., E. coli, Actinomyces meteri, Fusobacterium, Eubacterium lertum, and S. oralis were only found in samples from untreated canals. S. salivarius, P. endodontalis, A. odontolyticus, and Peptostreptococcus provetti were almost equally found in both primarily and secondarily infected root canals. The prevalence of bacteria from the root canals evaluated is presented in Table 1.

**Table 1:** The prevalence of bacteria from the root canals evaluated

Species	Prevalence
Peptostreptococcus spp.	16%
Sereptococcus spp.	14.2%
Porphyromonas spp.	12.2%
Enterococcus faecalis	9.6%
Staphylococcus salivarius	8.6%
Prevotella spp.	8.1%
Lactobacillus spp.	7.1%
Actinoimyces spp.	7.1%
Candida albicans	3.6%
Veillonella spp.	2.5%
Eubacterium spp.	2.5%
Bacillus spp.	2%
Eschrishia coli	1.6%

## Discussion

The present study evaluated the microorganisms collected from primary and secondary infection of root canals. Microorganisms were found in 140 of all the canals evaluated in this study. In 6.7% of the evaluated samples, no microorganisms were found. Other studies using culture methods for endodontic samples have also shown canals without any microorganisms.^1,2,4^

Primary infection of root canals is the result of colonization of microorganisms in a necrotic pulpal tissue leading to a dysfunction of the pulp. Propagation of pulpal infection depends on factors such as number of bacteria, caries, trauma, and iatrogenic variables. ^8 , 11 - 13^ It has been shown that improper restorations, carious lesions, and enamel cracks produced during access cavity preparation provide access routes to large dentinal tissues in many cases, leading to pulpal infection. In the present study, 61 teeth had a necrotic pulp, 10 of which revealed radiographic signs of periapical lesions. The results of this study showed that among the samples, Gram positive bacteria (67.8%) are more prevalent in primary root canal infections. This is consistent with the results of previous studies indicating 69% facultative anaerobics and 70% Gram positive bacteria in the infected root canals.^10,11^ The two most prevalent species among the collected samples were Peptostreptococcus and Streptococcus spp. Black-pigmented bacteria including Prevotella and Porphyromonas spp. were also seen in primary pulpal infections. These results coincide with the findings of previous studies.^1,2,11^

Secondary infections are produced by microorganisms resistant to chemico-mechanical procedures or as a result of bacterial invasion from microleakage of coronal restorations.^6,7^

Of the 49 teeth treated endodontically in the present study, 19 had a weak obturation for more than two years with radiographic signs of apical periodontitis. The most prevalent microorganisms among the samples of secondary infection were Gram positive bacteria. E. faecalis isolates were the most prevalent species, which is consistent with the results of previous studies. ^8 - 10^ Other isolated species included A. odontolyticus, L. aerophilus, S. salivarius, S. sanguis, P. corporis, P. gingivalis, and P. odontoma.

The results of the present study indicate that root canal infection occurs as a result of multiple microorganism activity, dominated by Gram positive bacteria. Polymicrobial anaerobic infections were also found in the root canal. Regarding the secondary infection, E. faecalis was found to be the most prevalent species.
